# Perceived feedback and school belonging: the mediating role of subjective well-being

**DOI:** 10.3389/fpsyg.2024.1450788

**Published:** 2024-10-10

**Authors:** Xinyu Li, Yi-Lung Kuo, Thomas J. Huggins

**Affiliations:** ^1^Great Ormond Street Institute of Child Health, University College London, London, United Kingdom; ^2^Applied Psychology Programme, Department of Life Sciences, BNU-HKBU United International College, Zhuhai, China; ^3^Pillar of Cognitive Sciences, College of Education Science, The Hong Kong University of Science and Technology, Guangzhou, China; ^4^School of Psychology, University of Monterrey, San Pedro Garza Garcia, Mexico

**Keywords:** school belonging, perceived feedback, subjective well-being, mediation analysis, PISA 2018

## Abstract

**Introduction:**

This study examined the interplay between perceived feedback (PF), subjective wellbeing (SWB), and students’ sense of belonging to school (SBS). School belonging is a key factor for decisions regarding academic studies, and is usually impacted by PF. The current study explored whether SWB mediates the established relationship between PF and SBS.

**Method:**

This was achieved by applying a mediation model to PISA 2018 data from 12,058 students in four areas of China.

**Results:**

Perceived feedback positively affected students’ SBS (*β* = 0.26, *p* < 0.01); and that SWB partially mediated this relationship between PF and SBS (*β* = 0.47, *p* < 0.01).

**Discussion:**

The findings of this study have the potential to contribute to the existing literature on educational psychology and well-being. By shedding light on the mediating role of SWB, the research offers practical implications for educators and policymakers seeking to enhance students’ school belonging and other implications for their academic success. Further research can contribute to this promising area, by focusing on subjective wellbeing and its impact on a range of decisions being made by students during a critical phase of their personal and academic development.

## 1 Introduction

### 1.1 Background

Middle school education is a key period for individual development and growth (ACT, 2008). According to Zhao et al. (2011), this phase includes much of an individual’s physical development, philosophical maturity, the consolidation of life values, and is also known as the *golden period*. This period of education constitutes the transition stage of adolescence, where students face many physical, emotional, and cognitive challenges (Csikszentmihalyi, 2024). Adolescence as a whole brings emotional fluctuations that may include emotional problems such as anxiety and depression (Armitage et al., 2024). At the same time, academic studies have become progressively more difficult and subjects have become more diverse. Students face more learning tasks, examination pressure, and competition, contributing to the emergence of various psychological disorders. According to global statistics, 1 in 7 (14%) adolescents aged 10–19 suffer from mental health problems (World Health Organization, 2021).

Belonging is a basic human emotional need. School belonging is defined as “the extent to which students feel personally accepted, respected, included, and supported by others in the school social environment” (Goodenow and Grady, 1993, p. 60). Associated research has indicated that a sense of belonging is a prerequisite for motivation (Baumeister and Leary, 1995) and that a high sense of belonging leads to stronger motivation (Renninger and Hidi, 2019, p. 118). School belonging is associated with positive outcomes such as psychosocial health, pro-social behavior, academic achievement, and successful transition into adulthood (Allen et al., 2021c). According to Ruvalcaba et al. (2017), this may be particularly valuable during the challenges posed by adolescence. In the absence of a sense of belonging, if an individual’s group does not match their values or interests and they are therefore not welcome or appreciated, this can lead to distress and disappointment (Dutcher and Quinn, 2023).

Belongingness research is broadly relevant to educational psychology (Allen et al., 2021a) – especially where a growing number of students around the world report not feeling a sense of belonging to their school (Allen et al., 2021c). The OECD (2019a,b) reports that one-third of students globally do not feel a sense of belonging to their school, and this number is steadily increasing. In primary and secondary school, students can consciously abide by school rules and regulations and classroom norms. They can consider the overall situation for the collective’s benefit. However, it is undeniable that as students grow up, the same connection between individuals and the collective can be severely challenged. Individual students can become very fragile and unstable, which can manifest itself in truancy, disinterest in collective activities, and the belief that there is no sense of security in the school. This argument is supported by research concluding that students’ truancy, lack of positive learning attitudes, and even poor academic performance are due to students’ lack of sense of belonging in school (Ramberg et al., 2019; Virtanen et al., 2021). The presence of a sense of belonging, especially school belonging, has a powerful long-term and short-term impact on students’ positive psychological and academic outcomes (Allen et al., 2021c). Strengthening students’ sense of belonging is therefore essential for their wellbeing and development.

Widely conceptualized as a basic human need, belonging is a complex process because it has multifaceted components, predictors, and outcomes (Allen et al., 2021b; Baumeister and Leary, 1995). The mechanisms for enhancing school belonging are still being explored. School belonging is generally associated with better academic functioning, better psychosocial and emotional health, and fewer mental health problems (Allen et al., 2017; Arslan, 2018; Arslan, 2021). School belonging can also be treated as a key outcome in and of itself. For example, it has been shown that norms, shared experiences, and feedback have an impact on school belonging (Walton et al., 2012). Teacher support, peer support, participation in school activities, and identification with school norms are the most important factors in creating a sense of school belonging (Qin and Wan, 2015). Teacher mentoring enhances students’ motivation, engagement, and belonging (Qin and Wan, 2015; Allen et al., 2021c). Teacher feedback, in particular, can improve both students’ well-being in school and their level of social acceptance (Suldo et al., 2009).

The general purpose of the current article is to investigate the relationship between perceived feedback, school belonging, and subjective well-being in adolescents. It aims to contribute to the understanding of how feedback experiences impact students’ sense of belonging within the school context. Besides, there are some specific purposes. Firstly, to examine the influence of perceived feedback (both positive and negative) on adolescents’ feelings of school belonging. Secondly, to explore the mediating role of subjective well-being in the relationship between perceived feedback and school belonging. Thirdly, to provide insights for educators and policymakers on how to enhance students’ sense of belonging through effective feedback practices.

### 1.2 Literature review

#### 1.2.1 The relationship between perceived feedback and sense of belonging to school

Feedback has long been recognized as an effective tool for student learning (Hattie and Timperley, 2007). Hattie and Timperley (2007) defined feedback as information from a subject (teacher, peer, book, parent, experience, etc.) regarding an individual’s performance or comprehension. According to Talib et al. (2015), educational feedback is characterized as an assessment of a student’s learning and success that, when communicated to the student, indicates their performance level. According to Chaudron (1988), teacher feedback is the main way for students to learn about their purpose and ability or other language behaviors, and it is also an effective resource for students to repair their discourse and improve their sense of purpose and confidence.

The main purpose of teacher feedback is to help students understand their academic performance and encourage them to make progress in their learning. Relevant feedback can be goal or task-oriented, specific, and neutral (Black and Wiliam, 1998) and that feedback should be given at an appropriate level (Hattie and Timperley, 2007). In addition to this, feedback should be timely, and the recipient of the feedback should be allowed to respond to the feedback and engage in a dialog with the provider, including encouragement as well as attention to the recipient’s control and self-esteem (Thurlings et al., 2013).

The term belongingness first appeared in the Hierarchy of Needs Theory developed by Maslow (1943). He believed that there are five basic human needs, which are arranged in a certain hierarchy from low to high namely physiological needs, safety needs, belonging and love needs, respect needs and self-actualization needs. After the physiological and safety needs are satisfied, people pursue the need for belonging and love. His explanation of belongingness is the individual’s desire to be emotionally connected to others and to belong to a group, to be accepted and have a place in that group, which includes giving love to others and receiving love from others.

According to Hurtado and Carter (1997), sense of belonging to school (SBS) is the psychological perception of being a part of a community or group. It is an intrinsic connection between an individual and the group to which he or she belongs (Allen et al., 2021a). An increased sense of belonging helps to improve an individual’s self-confidence, self-esteem, self-control, and sense of responsibility and, if this need is not met, people can feel lonely and isolated (Pedler et al., 2021; U-Thrive, 2023).

SBS is a particular feeling of acceptance, respect, and support from teachers and classmates in the school environment, and also a feeling of being an important part of school life and classroom activities, together with an emotional identification with the school (Goodenow, 1993). According to Bao and Xu (2006), SBS is defined as students’ intellectual, emotional, and psychological identification and commitment to the school they attend, their willingness to take on the duties and obligations of being a member of the school, and their willingness to participate in school activities.

Positive relationships between students and their teachers will predict a higher sense of school belonging, while teacher-induced worries will diminish students’ sense of school belonging (Qin and Wan, 2015). Research evidence suggests that strong student-teacher relationships promote a sense of belonging in school (Allen et al., 2021c). Besides, some studies have found that teachers’ welcoming, caring, and counseling also have an impact on school belonging (McMahon et al., 2008). Experienced teachers will promote school belonging in adolescents with Autism Spectrum Disorders (ASD) (Osborne and Reed, 2011).

Other research has demonstrated that student’s responses in the classroom are dependent on school identity. If positive feedback is provided in the classroom, students will develop a sense of belonging and identity, whereas inconsistencies between the school environment and the student’s personality will lead to negative feelings, hindering SBS (Faircloth and Hamm, 2005; Wallace et al., 2012). When students perceive their teachers to be warm and accepting and have a sense of social support that is responsive to caring, students are more likely to perceive themselves as academically competent and have a sense of school belonging (Hughes, 2011). Overall, when students feel cared for, accepted, and respected by their teachers, a good sense of school belonging is established.

#### 1.2.2 The relationship between PF and subjective well-being

In the mid-20th century, psychologists began to develop scales and questionnaires to measure subjective well-being. Diener (1984) proposed that an important measurement tool was the Subjective Happiness Scale. Wilson (1967) published the first review of research on subjective well-being, Correlates of Avowed Happiness. Since the 1990s, there has been an increasing amount of research on subjective well-being.

Subjective well-being refers to an individual’s cognitive evaluation of the overall quality of life-based on his or her criteria, including the evaluation of life satisfaction, negative emotions, and positive emotions in three parts, responding to the degree of life satisfaction (Diener, 1994). It can provide a comprehensive psychological indicator of individual’s quality of life, reflecting their social functioning and adaptability (Guo, 2019). Subjective well-being consists of people making various positive and negative evaluations of their lives and people’s emotional reactions to their experiences and is a general term for different assessments of life, events that occur, body and mind, and life circumstances (Diener et al., 2006).

Teacher support significantly influences adolescents’ subjective well-being (Tian et al., 2016; Zheng et al., 2022), with emotional support and instrumental support being the most influential types of teacher support in predicting SWB (Suldo et al., 2009). When students receive encouragement, praise, and recognition, they feel valued and affirmed, which increases self-esteem and self-confidence to promote subjective well-being. The manner and content of teacher feedback affect students in different ways. Positive, specific, and constructive feedback is more helpful to increase students’ learning motivation, and distinctly affirmative feedback plays a positive role in increasing students’ self-confidence, and their negative emotions (Allwright, 1984). On the contrary, negative or overly critical feedback may have a negative impact on students, making students feel shame and low self-esteem (Sutton, 2020). This means that feedback can have an impact on students’ well-being, depending on its positive or negative valence. Positive feedback can reduce students’ anxiety because they know that they have achieved something academically, making them feel happier and more fulfilled. It reduces the stress levels, frustration, and anxiety that students experience in solving tasks, which in turn stimulates more communication (Zhang, 2008). Considering the above, the current research hypothesized that there is a significant positive correlation between teachers’ academic feedback and secondary school students’ subjective well-being.

#### 1.2.3 The relationship between SWB and SBS

As outlined earlier, school belonging is a sense of emotional connection and belonging to the school community which is closely related to student’s SWB. There is a significant positive relationship between students’ subjective well-being and their sense of belonging in school (Chen et al., 2011), meaning that SWB can have a positive impact on SBS. When a student’s subjective well-being is strong, he or she is more likely to experience a strong sense of connection and belonging to others. It makes them feel like important and valued members of the school. Similarly, Xu and Fang (2021, p. 8) also pointed out that school bullying not only directly hinders secondary school students’ access to subjective well-being, but also indirectly diminishes their SBS by disrupting their SWB. According to research by Arslan (2021), students’ academic performance, psychological health, and social connections all have a positive relationship with a sense of school belonging. Adolescents with positive emotions may be better at focusing on the positive aspects of their surroundings, thereby increasing the perceived helpfulness of others in the school environment (Ruvalcaba-Romero et al., 2017). This may enhance their sense of belonging in school. The current research hypothesized that subjective well-being is related to school belongingness.

#### 1.2.4 The program for international student assessment

The Program for International Student Assessment (PISA) is an ongoing program initiated by the Organization for Economic Co-operation and Development (OECD), which is an international organization that aims to foster equality, opportunity, and well-being for people around the world. OECD also supports other education-related research, such as the Survey of Adults Skills (PLAAC), Education at a Glance, and Teaching and Learning International Survey. All these data can be accessed free of charge, on the official website of the OECD.

The PISA examination is administered to 15-year-old teenagers every 3 years, to measure their reading, mathematics, and science knowledge, together with their ability to deal with daily life challenges (OECD, 2021), the student’s background information is also collected, including: age, gender, and ethnicity. The reason why PISA chose 15-year-old adolescents is that in most OECD-administered regions, teenagers in 15-year-old are nearing the end of compulsory education. This means that the random selection of students and schools can be more inclusive of students with diverse socio-economic backgrounds (OECD, 2021).

The current research uses the latest available data on PISA, which was conducted in 2018. PISA 2018 was administered to more than 600, 000 students from 79 different OECD regions, and is considered to represent 32 million students in statistical terms. In addition to that, PISA2018 also offers additional surveys for OECD countries and economic zones to choose from, including the Information and Communication Technology (ICT) Familiarity Questionnaire, the Well-Being Questionnaire, the Financial Literacy Questionnaire, and the Educational Career Questionnaire for students. Further questionnaires are also available for teachers to respond to.

#### 1.2.5 The PISA in the Chinese mainland (“China,” in this study, for the sake of brevity)

China has participated in the PISA four times. Shanghai, China participated in PISA as an independent partner economy in 2009 and 2012 (OECD, 2009, 2012). In 2015, Beijing, Shanghai, Jiangsu Province, and Guangdong Province are considered as a representative of China, written as “Beijing-Shanghai-Jiangsu-Guangdong” (B-S-J-G) (OECD, 2015). Beijing, Shanghai, Jiangsu Province, and Zhejiang Province participated in PISA 2018 to represent China, written as “Beijing-Shanghai-Jiangsu-Zhejiang” (B-S-J-Z) (OECD, 2018).

**Figure 1:**
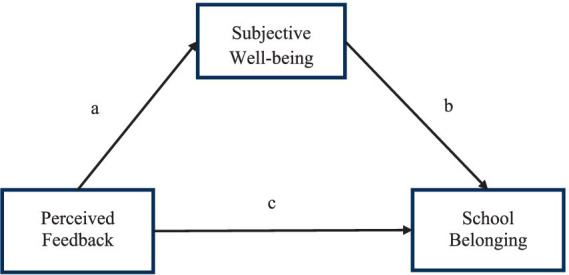
Proposed model for the role of subject well-being between perceived feedback and sense of belonging to school. Pathway a refers to the relation of Perceived Feedback to Subjective Well-being; Pathway b refers to the relation of Subjective Well-being to School Belonging; Pathway c refers to the relation of Perceived Feedback to School Belonging.

At the time of writing, it appears that no prior research has analyzed the relationships between PF, SWB and SBS, among students from this geographical area. Informed by the results of previous studies in other areas of the world, the current research is focused on filling this gap in academic knowledge. The sum of hypotheses outlined above combine to constitute the hypothetical mediation model shown in Figure 1. In sum: Hypothesis 1 - PF will significantly positively predict SBS; Hypothesis 2 - PF will significantly positively predict SWB, Hypothesis 3 - SWB mediates between PF and SBS.

## 2 Materials and methods

### 2.1 Data and participants

The data for this study were collected from PISA, including questionnaires completed by 15-year-old students. According to OECD (2019a,b), PISA samples are subjected to a rigorous sample selection process using the two-stage stratified sampling method to ensure a representative sample. The sampling probability was proportional to the number of expected 15-year-old students in the school. The sample sampling process was divided into two stages, stage one: selection of schools. In this stage, a certain number of schools were randomly selected from the list of schools and at least 150 were selected. Stage 2: Selection of students. A certain number of students were randomly selected from the selected schools to ensure diversity in the sample. Participants were randomly chosen from middle schools in Jiangsu Province, Zhejiang Province, Shanghai, and Beijing in China (OECD, 2018). A total of 12,058 samples (5,775 females; 6,283 males) from 361 different schools were drawn from the PISA 2018 B-S-J-Z database, with an average age of 15.75 years old (*SD* = 0.30). The distribution of the student sample is shown in Table 1. The current research study used the listwise method to eliminate invalid data, whenever single data was invalid, the questionnaire results for that sample were deleted. Finally the study obtained a valid sample of 11,584 with an effective rate of 96.07%. Among the responses retained, 6,025 (52%) were from male students and 5,559 (48%) were from female students. The total number of participants after data weighting is 950, 908.

**Table 1:** Distribution of samples in China.

	Male	Female	Student population	Number of schools
B-S-J-Z	6,283 (52.1%)	5,775 (47.9%)	12,058	361

B-S-J-Z = Beijing, Shanghai, Jiangsu Province, and Zhejiang Province.

### 2.2 Measures

#### 2.2.1 Instruments

All three questionnaires were created by assess developer. PISA2018 questionnaire framework was developed by the questionnaire expert group at John de Jong and Christine Rozunick from Pearson. The questionnaire expert group was chaired by Fons van The Questionnaire Expert Group was led by Fons van de Vijver. Other experts who contributed to the development of the questionnaire framework were Dominique Lafontaine, Sarah Howie, Andrew Elliot, Therese Hopfenbeck and David Kaplan (OECD, 2019a,b, p. 4).

#### 2.2.2 Perceived feedback

The PISA 2018 Student Background Questionnaire uses three questions to measure students’ Perceived Feedback, all of which are on a four-point scale consisting of three questions that are continuous variables. In order, they are “The teacher gives me feedback on my strengths in this subject,” “The teacher tells me in which areas I can still improve,” and “The teacher tells me how I can improve my performance.” The options include four categories, namely “Never or almost “, “Some Lessons,” “Many lessons,” and “Every lesson or almost every lesson,” each of which is worth 1 to 4 points, respectively. The higher the students’ scores on all topic responses, the stronger the perceived teacher feedback, indicating more positive teacher feedback. The results of this survey showed that the reliability of this scale was good with an internal consistency coefficient of 0.73 based on the current study participants.

#### 2.2.3 Subjective well-being

The investigation is focused on students’ subjective well-being, in terms of the emotions reported in the PISA, including nine items. The PISA categorizes students’ feelings into five positive emotions: “Happy,” “Lively,” “Proud,” “Joyful,” “Cheerful “; and four negative emotions: “Scared,” “Miserable,” “Afraid,” and “Sad.” The options consisted of four categories, namely “Never,” “Rarely,” “Sometimes,” and “Always “, each assigned a respective score of 1 to 4. As with the school belongingness treatment questions, this study reversed the scoring treatment for the items regarding negative experiences. As a result, higher scores on all responses indicate a higher level of SBS. The results of this survey showed that the reliability of this scale was good with an internal consistency coefficient of 0.83 based on the current study participants.

#### 2.2.4 The sense of school belonging

In PISA 2018, the Questionnaire used six questions to measure students’ sense of belonging to the school, all of which were based on a four-point scale. This scale is divided into two main dimensions, positive feelings, and negative feelings, and is used to measure secondary school students’ SBS as a whole. These positive feelings were included in the questionnaire: “I make friends easily at school,” “I feel like I belong at school,” and “Other students seem to like me.” The negative feelings presented in the questionnaire were “I feel like an outsider (or left out of things) at school,” “I feel awkward and out of place in my school,” and “I feel lonely at school.”

All questions on the PISA 2018 School Belonging Questionnaire range from “Strongly Agree” to “Strongly Disagree,” with scores ranging from 1 to 4, respectively. As with the prior two scales, items regarding negative sentiments are reverse coded. The survey’s findings demonstrated a good reliability for the SBS scale, with an internal consistency coefficient of 0.83 based on the current study participants.

### 2.3 Data analytic strategy

Quantitative methods were used in this study. Due to the sampling method used by PISA, both remaining students’ and schools’ data were weighted accordingly by SPSS 26.0 W_FSTUWT, a weight variable provided by PISA. This variable is recommended by the OECD to reduce biases caused by random sampling and for obtaining unbiased parameter estimates and standard errors. After being weighted by W_FSTUWT, the original set of 11,584 participating students represent 950,908 students, including 455,339 females (47.9%), and 495,569 males (52.1%). The subsequent analysis used SPSS 26.0 for data management and statistical analysis such as descriptive statistics and correlation analysis. The PROCESS plugin for SPSS was used for mediation model analysis. Bootstrap analysis was used to test the mediating effect of SWB between PF and SBS. According to Wen and Ye (2014), Bootstrap method has higher testing power than other methods to obtain more precise confidence intervals. It directly tests the significance of the product term of the regression coefficients and is useful in various complex models.

## 3 Results

### 3.1 Descriptive statistics and inter-correlations

Table 2 shows all variables’ mean, standard deviation, and correlation coefficients. Based on the descriptions, it can be seen that the means for PF, SBS, and SWB are 2.58, 2.95, and 2.82, respectively. Correlation analysis showed significantly positive relationships between all three variables: PF, SBS, and SWB.

**Table 2:** Descriptive statistics and correlations between key variables.

	*M*	*SD*	1	2	3
1. PF	2.58	0.88	–		
2. SBS	2.95	0.55	0.27^**^	–	
3. SWB	2.82	0.40	0.21^**^	0.50^**^	–

PF, Perceived feedback; SBS, Sense of belonging to school; SWB, Subject well-being. ^*^p < 0.05, ^**^p < 0.01.

### 3.2 Mediation effect analysis

For the purposes of the current analysis, PF constituted the independent variable, SBS was the dependent variable, and SWB was used as a mediator to analyze the mediating effect. The results indicate that SWB partly mediates the relationship between PF and SBS. These analytical results are shown in Table 3. The same table shows that the independent variable, PF, has a significantly influence on the dependent variable SBS (*β* = 0.26, *p* < 0.01). After adding the mediating variable of SWB, the independent variable PF impacts significantly on the mediating variable SWB (*β* = 0.21, *p* < 0.01). At the same time, PF both directly influences students’ SWB (*β* = 0.16, *p* < 0.01), and also indirectly influences SBS via mediation by SWB (*β* = 0.47, *p* < 0.01).

**Table 3:** Mediation model test for perceived feedback.

Predictor variables	Outcome variables	β	*t*	R-sq
PF	SBS	0.26	29.09^**^	0.07
PF	SWB	0.21	22.77^**^	0.04
PF	SBS	0.16	20.26^**^	0.28
SWB		0.47	58.79^**^	

PF, Perceived feedback; SBS, Sense of belonging to school; SWB, Subjective well-being.

*p < 0.05, **p < 0.01.

Bootstrap analysis was used to test the mediating effect of SWB between PF and SBS, as shown in Table 4. The results of Table 4 show that none of the Bootstrap confidence intervals contains zero, suggesting that the direct and indirect effects of SWB on PF are statistically significant. Following the mediation effect test procedure and associated criteria formulated by Wen and Ye (2014), SWB appears to play a mediating role in the relationship between PF and SBS, where the intermediate effect of this mediation was 38%.

**Table 4:** Bootstrap analysis of significance tests for mediating effects.

Pathway	Effect	LLCI	ULCI
Total effect	0.16	0.15	0.17
PF → SBS	0.10	0.09	0.11
SBS → SWB → PF	0.10	0.09	0.11

PF, Perceived feedback; SBS, Sense of belonging to school; SWB, Subject well-being; Effect, Unstandardized indirect effects; LLCI, Lower Limit of Confidence Interval; ULCI, Upper Limit of Confidence Interval. *p < 0.05, **p < 0.01.

### 3.3 Path model

**Figure 2:**
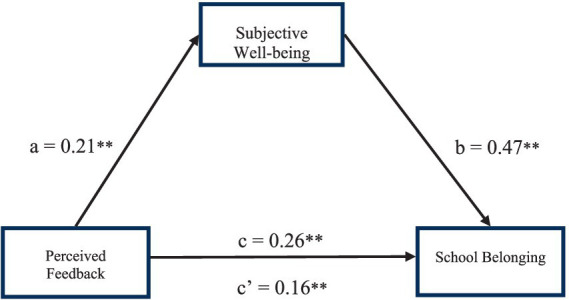
SWB mediation effect model. *p < 0.05, **p < 0.01.

Conclusions can be displayed in the pathway model shown in Figure 2. This model represents the relationships between the independent, mediator, and dependent variables. The standardized coefficients and effect significance are displayed for each relationship displayed.

## 4 Discussion

The results of this investigation indicated support for each of the three hypotheses: That PF influences SBS, that PF influences SWB, and that SWB mediates the relationship between PF and SBS. At the time of writing, these results constitute a novel mediating pathway for the relationship between PF and SBS which has not previously been analyzed among Chinese students. The current focus on secondary students makes these findings even more important, considering the crucial period of development that students in this age group tend to face.

Considering that PF was the only independent variable considered, these findings reinforce the importance of positive teacher feedback for the wellbeing of secondary students, as previously identified by Walton et al. (2012), Suldo et al. (2009), and by Qin and Wan (2015). It is clear that teachers need to use feedback to encourage secondary students. They can focus more on providing specific, positive feedback and targeted instruction, to establish a positive academic environment that promotes student commitment and development.

Where SBS is widely considered to be an important factor in secondary school students’ adjustment to school life and in the development of their social relationships (Pedler et al., 2021), the current research provides an important insight regarding ways to foster these benefits. The mediation effect outlined above provides robust support for promoting not only positive feedback, but also for focusing on the wellbeing of students. Where secondary students do not enjoy a good level of SWB, this is likely to dampen the positive impact of PF on the many benefits of SBS.

The latter finding has important implications for educators, school administrators, and policymakers aiming to improve the SBS of secondary school students. While SWB and other indicators of mental health can be hard to prioritize among other demands on secondary school administrators and teachers, the current study suggests that it is vital. As outlined above, a lack of SWB can disrupt the benefits that teacher feedback can have on students’ level of commitment to and identification with their secondary school institution. Improvements in SWB, through dedicated monitoring, support services, referrals, and programs aimed at mental health promotion, will maximize the benefits of positive teacher-student interactions.

### 4.1 Limitations and future research

Although the current research study presents information about the relationship between teacher feedback, subjective wellbeing and secondary school students’ sense of belonging to school, there are some limitations and potential research directions that have not been addressed. Among the limitations, the present study employed PISA 2018 as the original dataset to analyze. This dataset was largely constrained by PISA’s sampling method and structure. It was also published 5 years ago, meaning that the results may not be generalizable to students studying in 2024. Furthermore, the PISA B-S-Z-J dataset only included data from four regions in China. This means the results can only be strictly extrapolated to these four regions and are not representative of China as a whole.

In addition, the current research focused on cross-sectional data. Further research, using longitudinal datasets will be able to identify developmental changes. The combination of cross-sectional and longitudinal research may lead to even more discoveries, especially when this research includes a greater range of mental health indicators and cultural contexts. Further research would also benefit by considering how different forms of teacher feedback such as assessment, guidance, and praise can affect students’ subjective well-being. This would contribute to an even deeper understanding of how different educational strategies impact students’ psychological health and other educational outcomes.

## 5 Conclusion

In summary, the objective of the current research study was to test the relationship between PF and SBS, together with the mediating role of SWB. Decades of antecedents have shown the importance of each factor but few studies have combined them in a mediated model. The current results supported the existence of a positive relationship between PF and SBS. PF proved to be a highly significant predictor of SBS, meaning that when students feel more feedback from their teachers, they feel a greater sense of belonging to their school and class. The results also indicate that this relationship, between PF and SBS, is significantly mediated by SWB. This finding emphasizes opportunities for educational institutions to create supportive school environments, while also promoting the use positive academic feedback by all of their teachers.

In sum, educational institutions and policymakers need to commit to providing great school environments for fostering high levels of SWB and other aspects of mental health, based on the robust foundations of research into teacher feedback, positive student-teacher relationships, and other factors. Reinforcing high-quality teacher academic feedback in particular will help students improve both their subjective well-being and SBS. This will not happen by default or simply through a change in rhetoric, considering that many teachers have been educated through a lack of positive teacher feedback and may not have received applicable training or experiential opportunities. Dedicated training and even incentives may be required, together with student satisfaction monitoring and classroom observation. The current research supports these kinds of efforts to improve academic and developmental outcomes among secondary students.

## Data Availability

Publicly available datasets were analyzed in this study. This data can be found at: https://www.oecd.org/en/data/datasets/pisa-2018-database.html.
